# Enhancing *Morchella* Mushroom Yield and Quality Through the Amendment of Soil Physicochemical Properties and Microbial Community with Wood Ash

**DOI:** 10.3390/microorganisms12122406

**Published:** 2024-11-23

**Authors:** Kai Huang, Ling Li, Weijun Wu, Kunlun Pu, Wei Qi, Jianzhao Qi, Minglei Li

**Affiliations:** 1 Center of Edible Fungi, Northwest A&F University, Yangling 712100, China; 2 School of Soil and Water Conservation Science and Engineering, Northwest A&F University, Yangling 712100, China; 3 Shaanxi Key Laboratory of Natural Products & Chemical Biology, College of Chemistry & Pharmacy, Northwest A&F University, Yangling 712100, China

**Keywords:** *Morchella* yield, wood ash, microbial community, soil fertility

## Abstract

*Morchella* mushroom is a nutritionally rich and rare edible fungus. The traditional cultivation model, which relies on expanding the cultivation area to meet market demand, is no longer sufficient to address the rapidly growing market demand. Enhancing the yield and quality of *Morchella* without increasing the cultivation area is an intractable challenge in the development of the *Morchella* mushroom industry. Against this backdrop, this study investigates the effects of different amounts of wood ash (WA) application on the yield and quality of *Morchella*, and conducts an in-depth analysis in conjunction with soil physicochemical properties and microbial communities. The results indicate that the application of WA improves both the yield and quality of *Morchella*, with the highest yield increase observed in the WA2 treatment (4000 kg/hm^2^), which showed a 118.36% increase compared to the control group (CK). The application of WA also modified the physicochemical properties of the soil, significantly improving the integrated fertility index of the soil (IFI, *p* < 0.05). The soil microbial community structure was altered by the addition of WA. Redundancy analysis (RDA) revealed that pH and total potassium (TK) were the main environmental factors influencing the bacterial community, while pH, TK, and total nitrogen (TN) were the main factors influencing the fungal community structure. In addition, bacterial community diversity tended to increase with higher WA application rates, whereas fungal community diversity generally showed a decreasing trend. Furthermore, the relative abundance of beneficial microbial communities, such as *Acidobacteriota*, which promote the growth of *Morchella*, increased with higher WA application, while the relative abundance of detrimental microbial communities, such as *Xanthomonadaceae*, decreased. Partial least squares path model (PLS-PM) analysis of external factors affecting *Morchella* yield and quality indicated that WA application can alter soil physicochemical properties and soil microbial communities, thereby improving *Morchella* yield and quality. Among these factors, soil fertility was identified as the most important determinant of *Morchella* yield and quality.

## 1. Introduction

*Morchella*, commonly known as morel mushroom, belonging to the family Morchellaceae [1], is one of the few rare edible fungi in the Ascomycota that can be cultivated artificially. It is named after the concave surface of its fruiting body, which resembles a sheep’s stomach [2]. *Morchella* has an excellent taste, delicious flavour and is rich in nutritional components, making it of high economic and nutritional value [3,4]. Since the commercial cultivation of *Morchella* was realised in China in 2012, the industry has grown from an initial 200 hectares to 16,466 hectares by 2022 due to its low labour intensity, short cycle, and high profits [5]. In just ten years, production has increased more than 80-fold and cultivation has rapidly expanded to more than 20 provinces nationwide [5]. However, as the industry continues to develop, some problems have begun to emerge, such as the highly unstable yield and quality of *Morchella* [6,7]. There is an urgent need for in-depth research into the growth mechanisms of *Morchella* to find methods to increase and maximise its yield.

*Morchella* exhibits a preference for burned soil, with high-altitude fir and spruce forests producing a large crop of mushrooms in the year following a fire, with a gradual decline in yield in subsequent years [8]. Research has shown that the growth and development of *Morchella* requires a substantial supplement of potassium, and the use of WA can result in high yields of *Morchella* [9]. WA is not only an effective soil amendment [10], but also an abundant and inexpensive natural potassium fertiliser, containing significant amounts of potassium, phosphorus, and other nutrient elements [11]. When applied, it can regulate soil pH, improve soil fertility [12], increase pore space within soil particles, and improve soil water retention capacity [13]. In addition, WA has disinfectant and germicidal properties, helping to control and prevent diseases such as damping-off, wilt, and grey mould in crops [14]. Like wood ash, biochar can improve soil structure and microbial communities, promote plant root growth, and enhance plant stress and disease resistance [15,16]. However, biochar may produce some toxic by-products during the production process, which may have a negative impact on plants and soil microorganisms [17].

Soil microorganisms, as key decomposers, are integral components of the soil ecosystem and play a key role in improving soil structure, regulating soil nutrient balance, and influencing crop yield [18]. In addition, microorganisms are sensitive biological indicators of changes in the soil environment, and soil microbial diversity and community structure can serve as important indicators for assessing soil fertility [19,20]. The application of plant ash can increase pH levels, thereby affecting the microbial communities within the peat layer [21]. In addition, studies have shown that the addition of plant ash can increase the abundance of actinobacteria, which play an important role in the decomposition of soil organic matter, thereby influencing plant use of soil organic matter [22]. The effects of plant ash on microbial activity include changes in the composition, abundance, and diversity of microbial communities, with soil pH and concentrations of key soil nutrients being the main drivers of these changes [23,24,25].

As an effective soil amendment, plant ash can not only directly affect crop yield and quality by altering soil physicochemical properties [10,11,12,13], but also improve soil nutrient use efficiency by altering microbial community structure, thereby promoting crop growth and development [21]. However, there are few reports on the cultivation of *Morchella* with the addition of plant ash, and the micro-mechanisms by which plant ash enhances *Morchella* yield remain unclear, thus failing to provide a scientific basis for *Morchella* cultivation. Therefore, as shown in the flowchart of this experiment in Figure S1, this experiment aims to clarify the relationship between *Morchella* yield, soil physicochemical properties, and microbial communities by comparing the effects of different amounts of plant ash on *Morchella* yield and quality, soil physicochemical properties and soil microbial communities. The aim is to provide scientific guidance for the selection of the optimal amount of plant ash to be added to *Morchella* cultivation.

## 2. Materials and Methods

### 2.1. Overview of Experimental Site and Materials

The strain of morel mushroom used in this experiment was *Morchella sextelata*, with NGBI GenBank accession number PQ527894, and its specimen is deposited at the Centre for Edible Mushrooms of the Northwest Agriculture and Forestry University under accession number FC202201. The experimental site is located in the trial fields of the Edible Fungus Centre, Northwest A&F University, Yangling District (coordinates: 108.08° N, 34.28° E), Xianyang City, Shaanxi Province, China, at an altitude of 458.7 m. The climate of the region is characterised as a continental monsoon semi-humid climate with distinct seasons, an average annual temperature of 12.9 °C, an annual rainfall ranging from 635.1 to 663.9 mm, and an average sunshine duration of 2163.8 h. 

In this experiment, a cultivation method was used that mimicked forest conditions, and the experimental plot was equipped with irrigation facilities. Before tillage, the physicochemical properties of the 0–30 cm thick soil layer were as follows: pH 8.32, soil bulk density (BD) 1.28 g/cm^3^, soil moisture content (SMC) 21%, organic matter content (SOM) 14.14 g/kg, total nitrogen content (TN) 0.81 g/kg, total phosphorus (TP) 0.69 g/kg, total potassium (TK) 20.29 g/kg, available phosphorus (AP) 11.47 mg/kg, rapidly available potassium (AK) 125 mg/kg, and alkali-hydrolysable nitrogen (AN) 47.93 mg/kg.

The WA used in this study was obtained from Baoji Shengli Modern Agricultural Development Co., Ltd., Baoji, China. The physicochemical properties of WA are as follows: pH of 9.38, organic matter content (OM) of 9.81 g/kg, total nitrogen content (TN) of 0.55 g/kg, total phosphorus content (TP) of 4.03 g/kg, total potassium content (TK) of 37.23 g/kg, available phosphorus content (AP) of 917.32 mg/kg, rapidly available potassium content (AK) of 6400 mg/kg and alkali-hydrolysable nitrogen content (AN) of 221.91 mg/kg.

### 2.2. Experimental Design and Pretreatment of Soil Microbial Samples

The experimental design was divided into five treatment groups: (1) CK, no application of WA; (2) WA1, application of 2000 kg of WA per hectare; (3) WA2, application of 4000 kg of WA per hectare; (4) WA3, application of 6000 kg of WA per hectare; and (5) WA4, application of 8000 kg of WA per hectare. Each plot measured 3 × 3 m, with three replicates for each treatment group. A drainage ditch 80 cm wide and 25 cm deep was installed between adjacent plots to reduce cross-contamination. The key aspects of the cultivation process are displayed in Figure S2, and all cultivation practises followed standard agricultural practises.

Sampling was carried out during the ripening phase of the morel mushrooms. Within each plot, 20 fruiting bodies with similar growth characteristics and that were free of disease were randomly selected to measure their horticultural characteristics. Soil samples were taken from a depth of 5–10 cm, in a radius of 3 cm around the base of the selected fruiting bodies. The soil was then sieved through a 2 mm mesh to remove debris and the samples were divided into two aliquots (5 g each). One aliquot was stored at 4 °C for analysis of soil physicochemical properties, while the other was stored at −80 °C for analysis of microbial communities.

### 2.3. Determination of Fruiting Body Indices and Soil Physicochemical Properties

The method for determining soil physical and chemical properties is as follows [26]: The soil pH value is measured by shaking a pH metre in a suspension of soil/water (1:2.5) for 30 min. The alkaline nitrogen content (AN) is determined by diffusion method. The content of available phosphorus (AP) was determined by the sodium bicarbonate extraction molybdenum antimony anti-spectrophotometric method. The soil organic matter content (SOM) was determined using potassium dichromate oxidation spectrophotometry. Quick-acting potassium (AK) was determined by the 1.0 mol·L^−1^ extraction flame photometry method. The total nitrogen content (TN) of the soil was determined using the semi-trace Kjeldahl method. The total phosphorus content (TP) of the soil was determined by sulfuric acid perchloric acid digestion molybdenum antimony anti-spectrophotometry; The total potassium content (TK) of the soil was determined using the continuous flow method.

### 2.4. Extraction and Sequencing of Soil Microbial DNA

The soil DNA extraction kit (DP812, TGuide S96 magnetic bead method) from Tiangen Biochemical Technology Co., Ltd. (Beijing, China) was used for DNA extraction. PCR amplification was performed using qualified DNA as a template. The upstream primer sequence for amplifying the V3–V4 high variable region of bacterial 16S rRNA was 341F: 5′-CCTAYGGGRBGCASCAG-3′, and the downstream primer sequence was 806R: 5′-GACTACHVGGGTWTCTAAT-3′ [27]. The upstream primer sequence for amplifying the ITS1 region of the fungal ITS gene is 1737F: 5′-CTTGTCATTTAGGGAAGTAA-3′, and the downstream primer sequence is 2043R: 5′-GCTGTTCTATCGATGC-3′ [28]. PCR amplification system: pre-denature at 95 °C for 3 min; 35 cycles at 95 °C for 30 s, 50 °C for 30 s, and 72 °C for 1 min; extend at 72 °C for 10 min and end the reaction at 4 °C. Detect, recover, and purify PCR reaction products, and send qualified samples to Yangling Youruijie Biotechnology Co., Ltd. (Xianyang, China). Sequencing was performed on the Illumina MiSeq PE300 platform to generate raw data. The DADA2 pipeline was used for primer removal, quality filtering, denoising, chimaera removal, and contig assembly. The tag sequence is annotated with a species database (Silva database, https://www.arb-silva.de/ (accessed on 1 July 2024) for 16S, Unite database, https://unite.ut.ee/ (accessed on 1 July 2024) for ITS) [29].

### 2.5. Data Analysis and Statistics

Randomly selected subsets of sequencing data from the samples were used to calculate alpha diversity indices. The number of sequences sampled was plotted against the corresponding index values to construct curves, thereby validating whether the sequencing depth had reached a plateau of saturation. For this experiment, data processing and statistical analysis were performed using IBM SPSS Statistics 25.0 software. One-way analysis of variance (ANOVA) was used to compare differences between treatments. Origin 2024 software was used to plot bar graphs for fruiting body yield, changes in relative abundance at the soil bacterial phylum level, box plots for microbial α-diversity, and Pearson correlation heatmaps between fruiting body nutritional components and soil environmental factors. The vegan, dplyr, ggcor, ggplot2, and ape packages in R 3.3.1 software were used to perform non-metric multidimensional scaling (NMDS) analysis based on the Bray–Curtis distance algorithm to determine differences in microbial β-diversity. The pheatmap package in R 3.3.1 was used for the Pearson correlation analysis and to plot heatmaps of environmental factors at the bacterial phylum level. 

## 3. Results

### 3.1. The Effect of Different Amounts of WA on the Yield and Quality of Morchella

**Figure 1:**
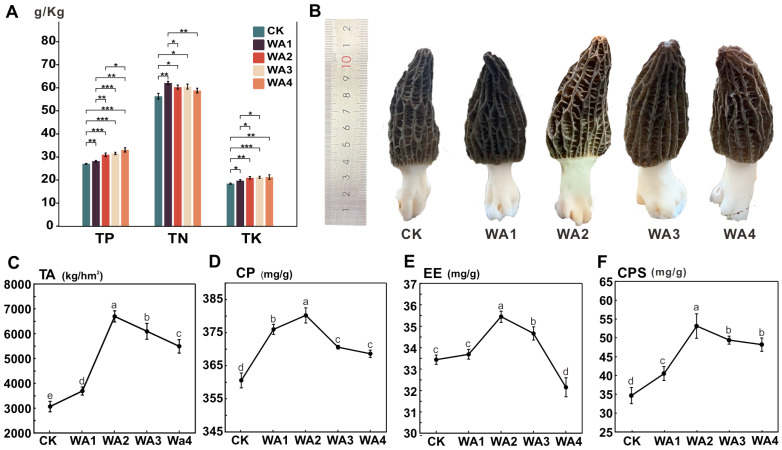
Yield (**A**), morphology (**B**), and nutrient composition (**C**–**F**) of the fruiting bodies of *Morchella* in different groups of WA additions. Different lowercase letters indicate significant differences (*p* < 0.05) in the same indicator under different vegetation types. * (0.01 ≤ *p* < 0.05), ** (0.001 ≤ *p* < 0.01), *** (*p* < 0.001).

The variation in the agronomic characteristics of *Morchella* under different WA treatments is shown in Figure 1 and Figure S2 and Table S1. The addition of different amounts of WA significantly affected the agronomic traits of *Morchella*. Intergroup ANOVA analysis showed that the yields of groups WA1 (3693.13 kg/hm^2^), WA2 (6696.53 kg/hm^2^), WA3 (6994.87 kg/hm^2^), and WA4 (5491.17 kg/hm^2^) were all significantly higher than that of the CK (3066.30 kg/hm^2^). In addition, the total yield (TA) of *Morchella* initially increased and then decreased with the increasing addition of WA, showing an increase of 20.44, 118.36, 98.77, and 79.08%, respectively, compared to the CK.

In terms of nutritional components, there were significant differences in crude protein (CP), crude fat (EE), crude polysaccharides (CPS), total nitrogen (FTN), total phosphorus (FTP), and total potassium (FTK) among the groups. In particular, groups P_2 and P_3 significantly increased the CP content, whereas group P_4 did not show a significant increase. The EE content in all treatment groups initially increased and then decreased, with groups P_2 and P_3 showing a significant increase, whereas group P_4 had an EE content of 3.22%, which was significantly lower than the CK. Compared to the CK, the CPS content was significantly higher in all treatment groups. The FTN content in groups P_1, P_2, P_3, and P_4 increased by 10.26, 7.03, 7.40, and 4.38%, respectively, with groups P_1, P_2, and P_3 showing significant increases compared to the CK. The FTP content in groups P_1, P_2, P_3, and P_4 increased gradually and was significantly higher than in the CK. The FTK content in groups P_1, P_2, P_3, and P_4 increased with the addition of WA and showed significant increases of 4.31, 14.61, 16.74, and 22.34%, respectively, compared to the CK.

**Figure 2:**
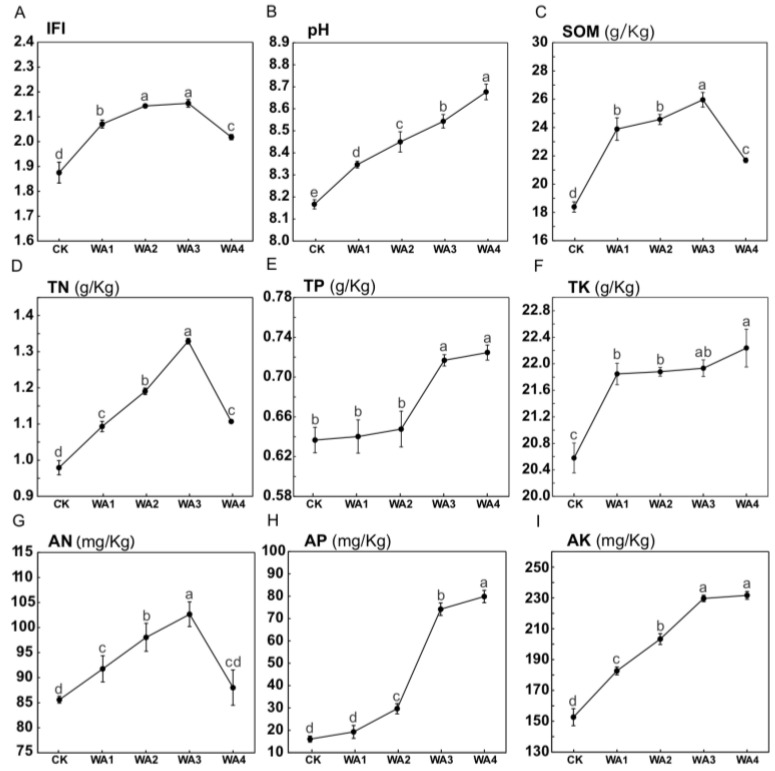
Variation in soil physicochemical properties. (**A**–**I**) shows the variation curves of integrated fertility index (IFI), pH, organic matter (SOM), total nitrogen (TN), total phosphorus (TP), total potassium (TK), alkaline-hydrolysed nitrogen (AN), available phosphorus (AP), and rapidly available potassium (AK), respectively. Different lowercase letters indicate significant differences (*p* < 0.05) in the same indicator under different vegetation types.

Considering the potential correlation between the enhancement of *Morchella* horticultural traits and soil fertility, the integrated fertility index (IFI) was determined. The IFI is a quantitative assessment method that integrates various soil physicochemical properties to provide a comprehensive reflection of soil fertility conditions. The primary indicators include pH, SOM, TN, TP, TK, AN, AP, and AK, among other soil physicochemical properties [30]. The different fertilisation treatments significantly affected the soil’s physicochemical properties (Figure 2, Table S2). As the amount of WA added increased, soil acidity (pH), AP, AK, TP, and TK showed a continuously increasing trend. Conversely, SOM, TN, and AN showed an initial increase followed by a decrease. The calculation method for IFI is shown in Table S3, and the results are shown in Table S4. With increasing application of WA, IFI showed a trend of initial increase followed by a decrease, showing a significant improvement compared to the CK. 

### 3.2. Microbial Community Diversity Analysis

Variation in soil IFI is thought to influence the structure of soil microbial communities. With this in mind, we investigated the effect of the addition of WA on the soil microbial community. The dilution curves for all sample groups flattened with increasing sequencing volume, indicating saturation of the sequencing data volume (Figure S3). According to the Venn diagram (Figure S4) and species composition heatmap (Figure S5) results, there are significant differences between different groups.

**Figure 3:**
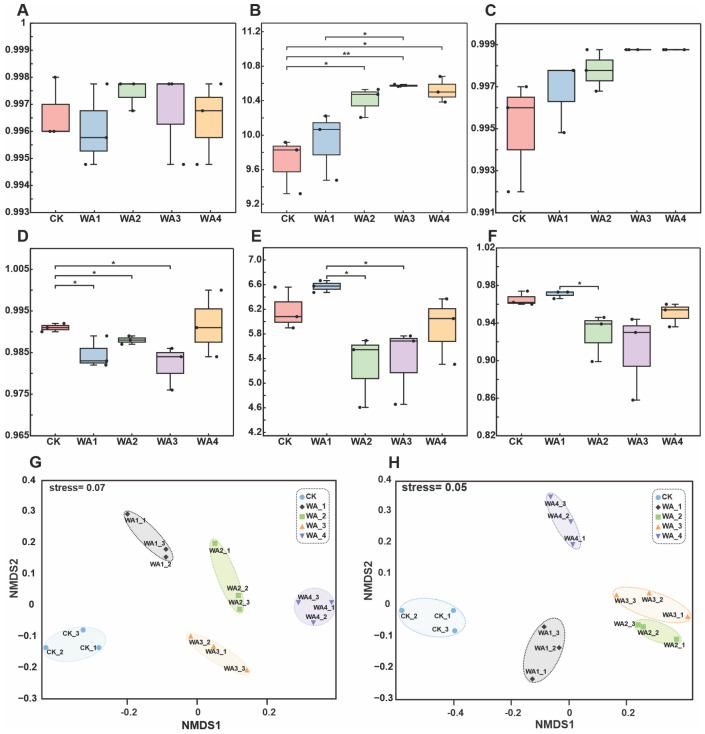
Microbial community diversity in different groups of WA additions. (**A**) The Goods_coverage index of the bacterial community. (**B**,**C**) The Shannon index and Simpson index of the bacterial community. (**D**) The Goods_coverage index of the fungal community. (**E**,**F**) The Shannon index and Simpson index of the fungal community. (**G**,**H**) NMDS analysis of bacterial community and fungal community. * (0.01 ≤ *p* < 0.05), ** (0.001 ≤ *p* < 0.01).

The bacterial Goods_coverage index for all sample groups was greater than 99%, indicating that the experimental sampling was appropriate and the results reliable, reflecting the true conditions in the soil (Figure 3A). Alpha diversity analysis showed that the Shannon index for bacteria was higher in each treatment group compared to the control group (CK) (Figure 3B). In particular, the P_3 group showed a significant increase compared to the CK (*p* < 0.01) (Figure 3B). Similarly, the Simpson index for bacteria was also higher in each treatment group than in the CK (*p* < 0.01) (Figure 3C). The Goods_coverage index for fungi was greater than 97% for all sample groups, indicating that the experimental sampling was adequate and the results reliable (Figure 3D). Alpha diversity analysis showed that the Shannon index (Figure 3E) and Simpson index (Figure 3F) for fungi in the P_2, P_3, and P_4 groups were lower than those in the CK. Although the P_1 group was higher than CK, there was no significant difference (Figure 3E,F). Overall, the relatively concentrated distribution of samples within groups suggests that the soil samples collected had good repeatability. Beta diversity analysis based on Bray–Curtis distances using NMDS showed stress values of 0.07 for bacteria (Figure 3G) and 0.05 for fungi (Figure 3H), indicating that the collected samples were well represented. The significant differences between the CK and the WA treatment groups suggest that the application of WA had a significant effect on the soil microbial community.

### 3.3. Microbial Community Compositional Variation Analysis

**Figure 4:**
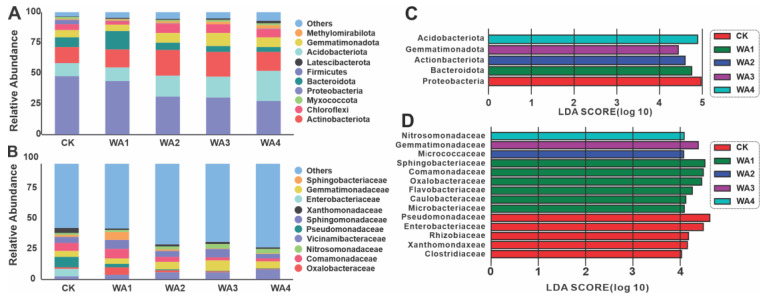
β-diversity analysis of bacterial communities. (**A**,**B**) Bacterial community composition at the phylum and family level in different treatment groups. (**C**,**D**) LEfSe elucidation of bacterial communities at the phylum and family level.

Subsequent analyses were directed towards examining the variations in the composition of microbial communities. The relative abundance of the top 15 phyla and families within each group was represented using stacked bar charts. At the phylum level, the mycorrhizal soil bacterial populations across the control and treatment groups are depicted in Figure 4A, successively identified as Proteobacteria, *Acidobacteriota*, *Actinobacteriota*, *Bacteroidota*, *Gemmatimonadota*, *Chloroflexi*, *Methylomirabilota*, and *Myxococcota* (with an average relative abundance of ≥1%). Despite Proteobacteria dominating in all treatment groups with proportions of 48.04, 44.09, 31.51, 30.72, and 27.75, respectively, there was a monotonic decrease in the relative abundance of Proteobacteria with an increment in the application of WA. In contrast, a gradual increase was noted in the relative abundance of *Acidobacteriota* and *Methylomirabilota*. The relative abundance of *Actinobacteriota* and *Bacteroidota* exhibited a biphasic trend, initially increasing and subsequently decreasing. To further elucidate the effects of varying quantities of WA on the structure of bacterial communities, a statistical analysis was performed on the predominant bacterial taxa at the family level. The analysis revealed a significant richness of families, including *Vicinamibacteraceae*, *Enterobacteriaceae*, *Oxalobacteraceae*, *Pseudomonadaceae*, *Gemmatimonadaceae*, *Sphingomonadaceae*, *Comamonadaceae*, *Sphingobacteriaceae*, *Nitrosomonadaceae*, and *Xanthomonadaceae* (with relative abundances of ≥1%), as shown in Figure 4B. Linear discriminant analysis effect size (LEfSe) elucidated the differential profiles among the various sample groups at distinct taxonomic levels. At the phylum level, significant disparities were observed between the treatment and control groups, as well as among the treatment groups, with the following taxa: Proteobacteria, *Acidobacteriota*, *Actinobacteriota*, *Bacteroidota*, and *Gemmatimonadota* (Figure 4C and Figure S4A). At the family level, the taxa that exhibited significant differences include *Nitrosomonadaceae*, *Gemmatimonadaceae*, *Micrococcaceae*, *Sphingobacteriaceae*, *Comamonadaceae*, *Oxalobacteraceae*, *Flavobacteriaceae*, *Caulobacteraceae*, *Microbacteriaceae*, *Pseudomonadaceae*, *Enterobacteriaceae*, *Rhizobiaceae*, *Xanthomonadaceae*, *Clostridiaceae*, and *Pyrinomonadaceae* (Figure 4D and Figure S4A).

**Figure 5:**
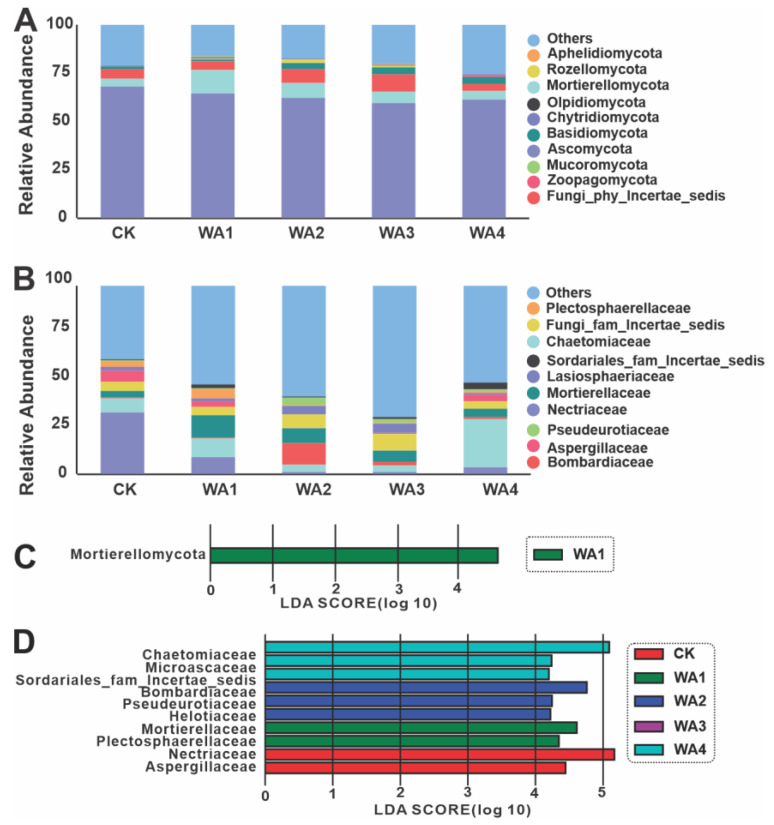
β-diversity analysis of fungal communities. (**A**,**B**) Fungal community composition at the phylum and family level in different treatment groups. (**C**,**D**) LEfSe elucidation of fungal communities at the phylum and family level.

Analysis of the fungal communities revealed that at the phylum level, the taxa with relative abundances ≥1% were, in order, *Ascomycota*, *Mortierellomycot*, *Fungi_phy_Incertae_sedis*, and *Basidiomycota* (Figure 5A). At the family level, the dominant fungal families were *Nectriaceae*, *Chaetomiaceae*, *Bombardiaceae*, *Mortierellaceae*, *Fungi_fam_Incertae_sedis*, *Aspergillaceae*, *Lasiosphaeriaceae*, *Plectosphaerellaceae*, *Pseudeurotiaceae*, *Sordariales_fam_Incertae_sedis*, *Helotiaceae*, and *Microascaceae* (Figure 5B). LEfSe analysis identified *Mortierellomycota* as the primary differential fungal phylum (Figure 5C and Figure S6B). The major differential fungal families were *Chaetomiaceae*, *Microascaceae*, *Sordariales_fam_Incertae_sedis*, *Bombardiaceae*, *Pseudeurotiaceae*, *Helotiaceae*, *Mortierellaceae*, *Plectosphaerellaceae*, *Nectriaceae*, and *Aspergillaceae* (Figure 5D and Figure S6B).

### 3.4. Redundancy Analysis (RDA) of the Correlation Between Soil Physicochemical Properties and Microbial Community

**Figure 6:**
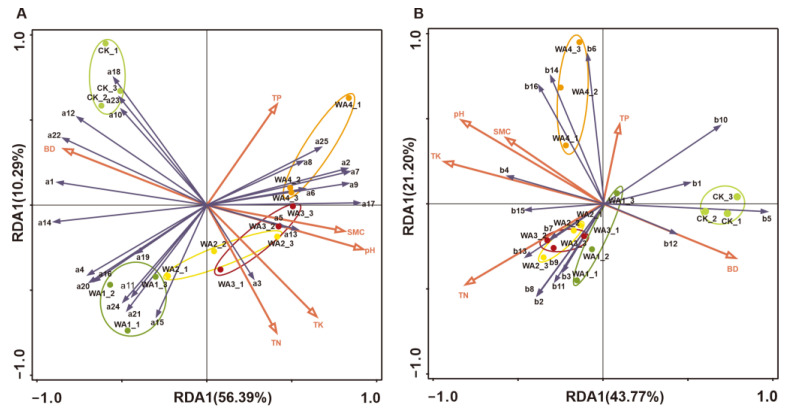
Combined RDA and Mantel tests analysis of bacterial (**A**) and fungal (**B**) communities. (**A**) the numerical labels represent bacterial taxa, specifically, a1: *Proteobacteria*, a2: *Acidobacteriota*, a3: *Actinobacteriota*, a4: *Bacteroidota*, a5: *Gemmatimonadota*, a6: *Chloroflexi*, a7: *Methylomirabilota*, a8: *Myxococcota*, a9: *Vicinamibacteraceae*, a10: *Enterobacteriaceae*, a11: *Oxalobacteraceae*, a12: *Pseudomonadaceae*, a13: *Gemmatimonadaceae*, a14: *Comamonadaceae*, a15: *Sphingomonadaceae*, a16: *Sphingobacteriaceae*, a17: *Nitrosomonadaceae*, a18: *Xanthomonadaceae*, a19: *Micrococcaceae*, a20: *Flavobacteriaceae*, a21: *Microbacteriaceae*, a22: *Rhizobiaceae*, a23: *Clostridiaceae*, a24: *Caulobacteraceae*, and a25: *Pyrinomonadaceae*. (**B**) the numerical labels represent fungal taxa, specifically, b1: *Ascomycota*, b2: *Mortierellomycota*, b3: *Fungi_phy_Incertae_sedis*, b4: *Basidiomycota*, b5: *Nectriaceae*, b6: *Chaetomiaceae*, b7: *Bombardiaceae*, b8: *Mortierellaceae*, b9: *Fungi_fam_Incertae_sedis*, b10: *Aspergillaceae*, b11: *Lasiosphaeriaceae*, b12: *Plectosphaerellaceae*, b13: *Pseudeurotiaceae*, b14: *Sordariales_fam_Incertae_sedis*, b15: *Helotiaceae*, and b16: *Microascaceae*.

In an effort to elucidate the relationship between soil physicochemical properties and microbial community composition, a redundancy analysis (RDA) was conducted to assess the correlation between environmental factors and microbial populations. Redundancy analysis of the relative abundance of key bacterial taxa and environmental factors accounted for 66.68% of the explained variation, with pH and total potassium (TK) contributing significantly to the variation in bacterial community composition at 48.5% (*p* < 0.05) and 13.3% (*p* < 0.05), respectively, highlighting them as two key environmental factors influencing bacterial communities (Figure 6A). Similarly, redundancy analysis of the relative abundance of key fungal taxa and environmental factors accounted for 64.97% of the explained variation, with total potassium (TK), total nitrogen (TN) and pH significantly contributing to the variation in fungal community composition at 39.5% (*p* < 0.05), 18.6% (*p* < 0.05) and 6.8% (*p* < 0.05), respectively, identifying them as three key environmental factors affecting fungal communities (Figure 6B).

**Figure 7:**
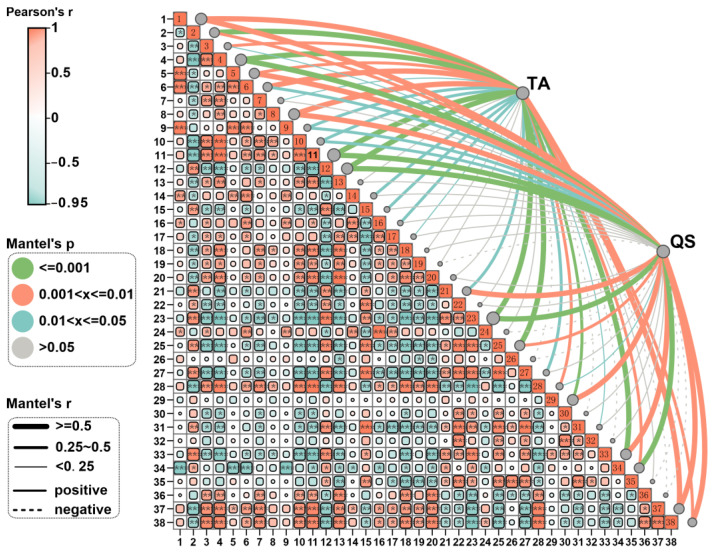
Mantel correlation analysis based on bacterial communities. 1: IFI, 2: BD, 3: SMC, 4: pH, 5: SOM, 6: TN, 7: TP, 8: TK, 9: AN, 10: AP, 11: AK, 12: *Proteobacteria*, 13: *Acidobacteriota*, 14: *Actinobacteriota*, 15: *Bacteroidota*, 16: *Gemmatimonadota*, 17: *Chloroflexi*, 18: *Methylomirabilota*, 19: *Xyxococcota*, 20: *Vicinamibacteraceae*, 21: *Enterobacteriaceae*, 22: *Oxalobacteraceae*, 23: *Pseudomonadaceae*, 24: *Gemmatimonadaceae*, 25: *Comamonadaceae*, 26: *Sphingomonadaceae*, 27: *Sphingobacteriaceae*, 28: *Nitrosomonadaceae*, 29: *Xanthomonadaceae*, 30: *Micrococcaceae*, 31: *Flavobacteriaceae*, 32: *Microbacteriaceae*, 33: *Rhizobiaceae*, 34: *Clostridiaceae*, 35: *Caulobacteraceae*, 36: *Pyrinomonadaceae*, 37: bacterial Shannon diversity index, 38: bacterial Simpson diversity index. * (0.01 ≤ *p* < 0.05), ** (0.001 ≤ *p* < 0.01), *** (*p* < 0.001).

To identify key microbial taxa closely associated with soil physicochemical properties, the Spearman correlation analysis was performed. The results indicated that AK was significantly positively correlated with *Acidobacteriota*, *Gemmatimonadota*, *Methylomirabilota*, *Vicinamibacteraceae*, *Gemmatimonadaceae*, *Nitrosomonadaceae*, and *Pyrinomonadaceae*, while showing significant negative correlations with *Proteobacteria*, *Bacteroidota*, *Enterobacteriaceae*, *Pseudomonadaceae*, *Comamonadaceae*, *Xanthomonadaceae*, *Flavobacteriaceae*, *Rhizobiaceae*, and *Clostridiaceae*. TK was significantly positively correlated with bacterial Shannon and Simpson indices, as well as *Nitrosomonadaceae*, and significantly negatively correlated with *Proteobacteria*, *Enterobacteriaceae*, *Pseudomonadaceae*, *Comamonadaceae*, *Xanthomonadaceae*, *Rhizobiaceae*, and *Clostridiaceae*. SOM was significantly positively correlated with the bacterial Shannon index, *Actinobacteriota*, and *Sphingomonadaceae*, while showing significant negative correlations with *Enterobacteriaceae*, *Pseudomonadaceae*, *Xanthomonadaceae*, *Rhizobiaceae*, and *Clostridiaceae*. AP was significantly positively correlated with *Acidobacteriota*, *Gemmatimonadota*, *Methylomirabilota*, and *Myxococcota*, and significantly negatively correlated with *Proteobacteria* and *Bacteroidota*. TN was significantly positively correlated with *Gemmatimonadaceae* and *Nitrosomonadaceae*, and significantly negatively correlated with *Enterobacteriaceae*, *Pseudomonadaceae*, *Comamonadaceae*, *Xanthomonadaceae*, *Rhizobiaceae*, and *Clostridiaceae* (Figure 7).

**Figure 8:**
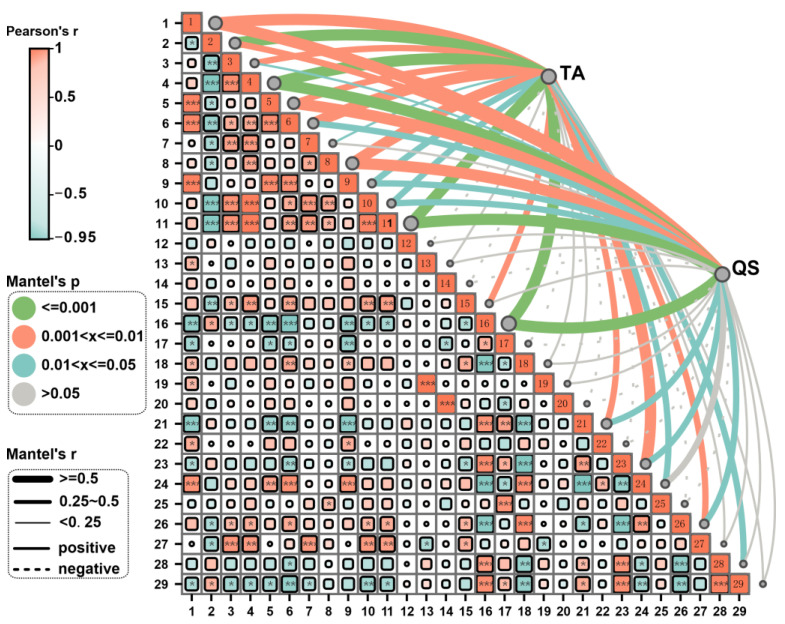
Mantel correlation analysis based on fungal communities. 1: IFI, 2: BD, 3: SMC, 4: pH, 5: SOM, 6: TN, 7: TP, 8: TK, 9: AN, 10: AP, 11: AK, 12: *Ascomycota*, 13: *Mortierellomycota*, 14: *Fungi_phy_Incertae_sedis*, 15: *Basidiomycota*, 16: *Nectriaceae*, 17: *Chaetomiaceae*, 18: *Bombardiaceae*, 19: *Mortierellaceae*, 20: *Fungi_fam_Incertae_sedis*, 21: *Aspergillaceae*, 22: *Lasiosphaeriaceae*, 23: *Plectosphaerellaceae*, 24: *Pseudeurotiaceae*, 25: *Sordariales_fam_Incertae_sedis*, 26: *Helotiaceae*, 27: *Microascaceae*, 28: fungal Shannon diversity index, 29: fungal Simpson diversity index. * (0.01 ≤ *p* < 0.05), ** (0.001 ≤ *p* < 0.01), *** (*p* < 0.001).

In the fungal community, AK was significantly positively correlated with Basidiomycota. AN was significantly positively correlated with *Fungi_phy_Incertae_sedis*, *Basidiomycota*, *Fungi_fam_Incertae_sedis*, *Lasiosphaeriaceae*, and *Pseudeurotiaceae*, and significantly negatively correlated with *Nectriaceae*, *Chaetomiaceae*, and *Aspergillaceae*. TK was significantly positively correlated with *Sordariales_fam_Incertae_sedis* and significantly negatively correlated with *Nectriaceae*. pH was significantly positively correlated with *Sordariales_fam_Incertae_sedis* and *Microascaceae*, and significantly negatively correlated with *Nectriaceae* and *Plectosphaerellaceae* (Figure 8).

### 3.5. Correlation Analysis Between the Yield and Quality of Fruiting Body and the Integration of Key Microorganisms and Soil Fertility

Following the establishment of the link between soil fertility and key microbial communities, further analysis of the association between fruiting body yield (TA) and quality (QS) and these factors was conducted: QS includes CP, where QS includes CP, EE, CPS, FTN, FTP, and FTK. The Mantel analysis revealed that TA exhibited significant correlations with *Proteobacteria*, *Actinobacteriota*, *Bacteroidota*, *Gemmatimonadota*, *Enterobacteriaceae*, *Pseudomonadaceae*, *Comamonadaceae*, *Sphingobacteriaceae*, *Nitrosomonadaceae*, *Xanthomonadaceae*, *Rhizobiaceae*, *Clostridiaceae*, bacterial Shannon and Simpson diversity indices, *Basidiomycota*, *Nectriaceae*, *Aspergillaceae*, *Plectosphaerellaceae*, *Pseudeurotiaceae*, and *Helotiaceae*, as well as fungal Shannon diversity (Figure 7 and Figure 8). QS showed significant correlations with *Proteobacteria*, *Enterobacteriaceae*, *Pseudomonadaceae*, *Comamonadaceae*, *Xanthomonadaceae*, *Rhizobiaceae*, *Clostridiaceae*, *bacterial Shannon and Simpson diversity indices*, *Nectriaceae*, *Aspergillaceae*, *Plectosphaerellaceae*, and *Helotiaceae*. The integrated fertility index (IFI) demonstrated significant correlations with *Proteobacteria*, *Pseudomonadaceae*, *Xanthomonadaceae*, *Rhizobiaceae*, *Clostridiaceae*, bacterial Shannon diversity, *Nectriaceae*, and *Aspergillaceae* (Figure 7 and Figure 8).

## 4. Discussion

### 4.1. Effects of Different Levels of WA Additions on the Yield and Quality of Morchella

The low yield and inconsistent quality of *Morchella* not only severely limit the further development of the morel industry, but also significantly dampen the enthusiasm of growers. This experiment investigated the effects of different amounts of WA application on the yield, quality, and physicochemical properties of the soil for *Morchella*. The results showed that the application of different amounts of WA significantly increased the yield of *Morchella*, with the WA2 group showing the most significant increase, which was 1.18 times that of the CK. In terms of quality, namely nutritional components, the general trend for crude protein and crude polysaccharides in *Morchella* was an initial increase followed by a decrease, and all treatment groups were higher than the CK. This trend may be related to the significant increase in soil pH caused by the addition of WA, as previous reports have indicated that an alkaline environment can inhibit the synthesis of crude protein and crude polysaccharides [31]. In this experiment, the trend for crude fat content was similar to that for crude protein and crude polysaccharides, with the growth rate compared to the CK ranging from −3.85% to 6.01%. The exception was the WA4 group, which had a lower crude fat content than the CK. A possible reason for this is that an excessively high pH significantly inhibits fat synthesis in the fungal bodies. As a green and readily available fertiliser, plant ash has the advantages of high efficiency and low cost. Taking 2022 as an example, the price of plant ash is CNY 170 per tonne, far lower than other fertiliser prices. Taking the WA2 group (4000 kg/hm^2^) as an example, the yield of morel mushrooms increased by 118.36%. Therefore, while increasing the yield of morel mushrooms, plant ash can also reduce production costs and increase the income of growers. However, experimental results show that moderate use of plant ash can improve soil structure and increase plant yield, but excessive use can lead to various negative effects mentioned above. From the experimental results, it can be seen that when the amount of plant ash added exceeds a certain threshold, the pH value of the soil significantly increases and plant growth is inhibited. Ash from plants and trees is rich in alkaline substances such as potassium and calcium. Excessive use can lead to an increase in soil pH, which may reduce the absorption of nutrients and water by crops, thereby affecting their growth and development [32]. Meanwhile, excessive potassium may inhibit the absorption of other essential elements, leading to nutritional imbalance [33]. Therefore, in the later stage of production, it is necessary to reasonably control the amount of wood ash added.

Research has shown that an adequate intake of K can help reduce the risk of high blood pressure in humans [34]. The K content in *Morchella* fruiting bodies is one of the key factors in evaluating the quality of *Morchella*. The content of TP and TK in *Morchella* is closely related to their growth and metabolic activities. The TN content can reflect the protein level of *Morchella* [35]. This study found that the application of WA can significantly (*p* < 0.05) increase the content of TK, TN, and TP in *Morchella*. Research has shown that wood ash is rich in nitrogen and phosphorus, and the application of wood ash can significantly increase the potassium content in the soil, thereby increasing the potassium and phosphorus content in plants [36]. However, the impact of wood ash on nitrogen is relatively complex. Some studies have shown that wood ash itself does not directly provide a large amount of nitrogen, but can improve soil pH and structure, enhance soil cation exchange capacity, and help plants better absorb nitrogen from the soil [25]. Moreover, the addition of elements such as nitrogen, phosphorus, and potassium can enhance microbial activity, thereby improving the plant’s ability to absorb nutrients [37].

### 4.2. The Effect of Different Amounts of WA on Soil Physicochemical Properties

As an effective soil amendment, WA is characterised by its loose and porous nature, which can significantly improve soil porosity and permeability. This improvement helps to increase the aeration and water retention capacity of the soil [38]. Our study found that the application of WA can reduce soil bulk density and increase soil water content (Table S2), providing a suitable growth environment for the moisture-loving and oxygen-requiring *Morchella* mycelium, which is consistent with previous reports [39]. The application of WA improves soil fertility due to its high content of alkaline elements such as K and P (Figure 2A). When applied to soil, it effectively raises soil pH [40] and increases the levels of N, P, K, and SOM [41,42]. Soil pH is a limiting factor for plant development and is closely related to soil nutrient dynamics and microbial activity [43]. *Morchella* perform better at pH 7.7 or above [44], and, in our study, we observed an upward trend in soil pH (8.2–8.7) with increasing amounts of WA, which meets the pH requirements for *Morchella*.

SOM is often closely related to many soil quality indicators and is one of the most important indicators of soil quality and productivity [45,46]. The application of WA increased the content of soil organic matter, total nitrogen, and ammonium N, but as the amount of WA applied increased, these indicators showed a decreasing trend in the WA4 group. Research indicates that when the amount of N applied exceeds the optimum N requirement, the residual inorganic N can accelerate the loss of soil organic matter and N by mineralisation [47,48]. This finding can explain the observed decline in soil fertility, organic matter, total nitrogen, and ammonium N in the WA4 group of our study. Therefore, the application of different amounts of WA can alter soil physicochemical properties and improve soil fertility. However, the differences in soil nutrient status and fruiting body growth under different application rates may be due to differences in soil microbial community structure.

### 4.3. Effects of Different Levels of WA Additions on Soil Microbial Community Structure

Microbial communities exert a significant influence within soil ecosystems, serving as the main catalysts for material and energy flows. Their diversity is a sensitive indicator of soil health [49,50], and the dominant microbial groups in the soil can vary with cropping patterns, fertiliser management, and crop growth stages [51,52,53]. The application of WA can significantly alter the structure of soil microbial communities and stimulate the activity of certain beneficial microbes, thereby indirectly improving crop stress resistance and yield [54,55,56]. This study found that the application of WA increased the Shannon and Simpson diversity indices for bacteria while decreasing those for fungi. Correlation analysis between microbial communities and soil physicochemical properties revealed a positive correlation between bacterial Shannon and Simpson indices and changes in pH and soil nutrient content (Figure 7). Research has shown that increasing bacterial community diversity can competitively inhibit the growth of pathogens and reduce the occurrence of plant diseases. Research shows that the diversity of the bacterial community is negatively correlated with the incidence rate of pathogens; that is, the higher the bacterial diversity, the lower the incidence rate of pathogens [57]. It is also possible to enhance plant stress resistance by producing plant growth regulators (such as plant hormones) and inducing systemic plant resistance, helping plants better cope with environmental pressures such as drought, salinity, etc. [58]. In contrast, fungal community diversity showed some negative correlation with the main soil physicochemical properties (Figure 8), as the decrease in fungal community diversity is mainly due to the application of plant ash, which leads to an increase in soil pH. A high-pH environment can inhibit the growth of some fungi [59]. Research has shown that a decrease in fungal diversity can indeed reduce competition, which is beneficial for the growth of *Morchella* [9,60]. These results suggest that WA application can alter the diversity of soil microbial communities by altering soil physicochemical properties. The diversity of microbial communities is highly sensitive to pH changes, with saline alkaline environments increasing the diversity of bacterial communities and decreasing the diversity of fungal communities [61].

Redundancy analysis has revealed that pH and total potassium (TK) are two significant environmental factors influencing bacterial communities, while TK, total nitrogen (TN), and pH are the three pivotal environmental factors affecting fungal communities. pH is one of the primary environmental factors shaping the structure of soil microbial communities, significantly altering the diversity and richness of bacteria and fungi [62]. WA, an alkaline particulate substance, can rapidly elevate soil pH upon incorporation into the soil [63]. Even an increase in pH of less than one unit is sufficient to significantly alter the growth environment of soil microorganisms [64]. N and potassium K, as nutrients essential for soil biological proliferation, can provide ample nourishment for microbes and enhance their capacity to utilise nutrient elements in the soil, which aligns with the findings of this study. Consequently, the application of WA, by increasing soil pH and the content of N and K, demonstrates that it can significantly impact soil microbial communities through the alteration of soil physicochemical properties.

This study shows that the application of WA can significantly influence the composition of soil microbial communities. At the phylum level, the application of WA increased the abundance of *Acidobacteriota*, *Actinobacteriota*, *Gemmatimonadota*, and *Methylomirabilota* compared to the CK, while reducing the abundance of *Proteobacteria*. *Acidobacteriota* play a role in various nutrient cycling processes in the soil, releasing nutrients that are available to plants [65]. It has been shown to have strong environmental adaptability, maintaining functionality under extreme conditions and influencing soil health [66,67]. In addition, *Actinobacteriota* play a crucial role in organic matter decomposition and nutrient cycling, and can effectively suppress the spread of various plant pathogens, with positive implications for agricultural production [22]. *Gemmatimonadota* are important in soil nitrogen and carbon cycling and are able to break down complex organic matter and improve soil fertility [68]. *Methylomirabilota* has attracted considerable interest in agricultural and environmental research for its functions and mechanisms of action, with studies suggesting that increasing its relative abundance can improve the efficiency of nitrogen fertiliser use [69].

The family *Gemmatimonadaceae* plays an important role in soils, especially in arid and semi-arid regions, where they make up a high proportion of the soil microbial community and may be involved in water and nutrient retention and cycling [70]. *Nitrosomonadaceae* can oxidise ammonia to nitrite, a key step in the nitrogen cycle, which in turn facilitates the utilisation of nitrogen by plants and other organisms [70]. In agricultural soils, the presence of *Nitrosomonadaceae* helps to improve soil fertility and crop productivity [71]. The family *Xanthomonadaceae* contains a number of damaging plant pathogens that infect plants in the family Cruciferae [72], as well as citrus [73] and sugarcane [74], thereby affecting crop yields and quality. The family *Clostridiaceae* likewise contains a number of disease-causing bacteria, whose increased abundance may negatively affect crop yields [75].

At the phylum level, the application of WA increased the abundance of *Mortierellomycota* and *Basidiomycota*, while decreasing that of *Ascomycota*. *Mortierellomycota* is an important component of soil fungal communities, with the ability to decompose organic matter and facilitate nutrient cycling in soils [76,77]. This group of fungi is considered beneficial, with fluctuations reflecting the health status of soil ecosystems [78]. *Basidiomycota* can improve plant resistance to disease and stress tolerance by engaging in mutualistic symbiotic relationships or acting as decomposers in the biogeochemical cycle [79]. At the family level, the addition of WA was observed to increase the abundance of *Bombardiaceae*, *Mortierellaceae*, and *Microascaceae*, while decreasing that of *Nectriaceae* and *Aspergillaceae*. *Mortierellaceae* and *Microascaceae* are integral members of soil fungal communities and play a crucial role in soil nutrient cycling, organic matter decomposition, and plant–microbe interactions. These microbial groups influence soil physicochemical properties through their unique metabolic pathways and ecological adaptability, thereby promoting plant growth, increasing crop yields, and contributing to ecosystem stability [80,81]. *Nectriaceae* have the potential to cause root diseases in plants and may also affect crop quality and yield through the production of toxins [82,83]. Fungi of the *Aspergillaceae* family, particularly some *Aspergillus* species, are known to colonise and damage a variety of crops including maize, peanuts, cottonseed, and nuts [84]. It can be further inferred that the application of WA may alter the physicochemical properties of the soil, thereby increasing the relative abundance of nutrient-rich groups and beneficial bacteria and decreasing the relative abundance of nutrient-poor groups and pathogenic bacteria in the soil. Ultimately, this could lead to an improvement in morel yield and quality. 

### 4.4. Effect of Soil Physicochemical Properties and Key Microbial Species on the Yield and Quality of Morchella

The results of the Mantel analysis (Figure 7 and Figure 8) show that both TA and QS of morels have a significant positive correlation with the IFI of the soil and are strongly influenced by the physicochemical properties of the soil. TA shows significant correlations with *Pseudomonadaceae*, *Rhizobiaceae*, *Nectriaceae*, *Plectosphaerellaceae*, *Comamonadaceae*, and bacterial Shannon diversity. QS is significantly correlated with *Proteobacteria*, *Enterobacteriaceae*, *Xanthomonadaceae*, *Rhizobiaceae*, *Clostridiaceae*, bacterial Shannon diversity, bacterial Simpson diversity, and *Gemmatimonadota*. *Pseudomonadaceae*, known for their plant growth-promoting and disease-suppressing properties, can increase crop yield by inhibiting harmful pathogens and promoting nutrient uptake. *Rhizobiaceae*, with their nitrogen-fixing properties, show a significant positive correlation with crop yield. Studies have shown that *Rhizobiaceae* increase the availability of nitrogen in the soil, thereby improving plant nitrogen availability and promoting yield improvement. *Nectriaceae* and *Plectosphaerellaceae* also show a highly significant positive correlation with crop yield, possibly by influencing the balance of soil microbial communities or promoting plant root health, thereby indirectly increasing crop yield. *Comamonadaceae*, which have a significant positive correlation with crop yield, play an important role in improving soil fertility and environmental restoration.

**Figure 9:**
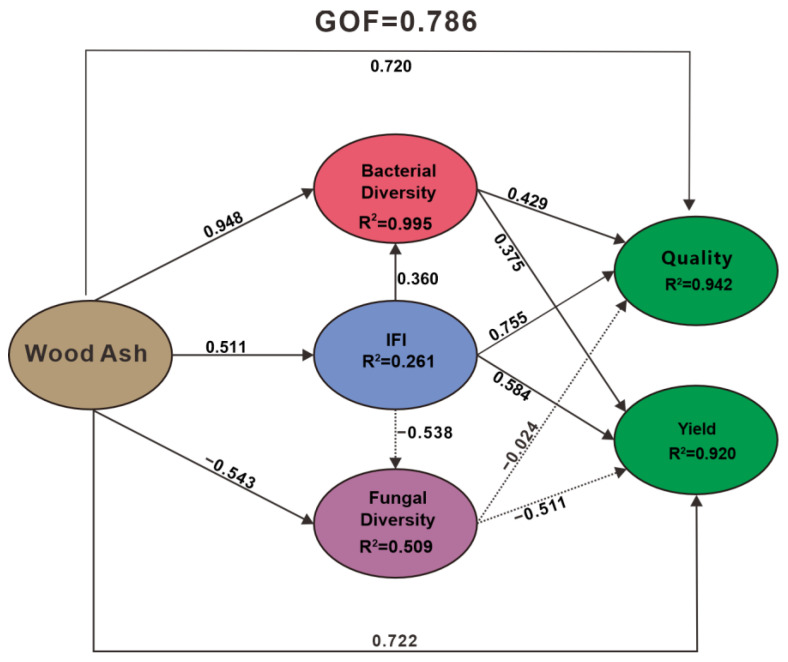
PLA-PM analysis of the main factors of quality and yield enhancement.

Compared to the CK, the application of WA has been found to enhance both the yield and quality of morels. By integrating the analysis of soil physicochemical properties and microbial community structure, this study postulates that both soil physicochemical properties and beneficial microbes (key microbial group) can play a positive role in the yield of morels. Partial least squares path modelling (PLA-PM) was employed to further discern the primary factors contributing to the increase in yield (Figure 9). The goodness-of-fit value of 0.786 indicates a high degree of model fit, suggesting that the model can reasonably explain and reflect the relationships within the data to a certain extent. The path coefficients between the addition of WA and bacterial community diversity (0.948), fungal community diversity (−0.543), and IFI (0.511) were determined. The path coefficients between bacterial community diversity, fungal community diversity, and IFI with morel yield were 0.375, −0.511, and 0.584, respectively. The total effects of bacterial community diversity, fungal community diversity, and IFI on QS were 0.429, 0.755, and −0.024, respectively. The addition of WA had a strong influence on bacterial community diversity (0.948), fungal community diversity (−0.543), and IFI (0.511), with the greatest impact on bacterial community diversity. Comparing the path coefficients of the three soil factors with yields TA and QS reveals that the yield and quality of morels are primarily influenced by soil factors. Notably, the path coefficient of IFI on QS reached 0.755, indicating that soil fertility plays a crucial role in QS. Additionally, the impact of soil fertility on morel yield is also the most significant (0.584). There is a strong negative correlation between fungal diversity and yield (−0.511), suggesting that the application of WA may increase yield while potentially reducing the abundance of harmful fungi in the soil. Overall, bacterial community diversity, fungal community diversity, and IFI all have varying degrees of influence on TA and QS, with IFI playing a key role. It can be inferred that the application of WA primarily enhances yield by altering soil physicochemical properties.

## 5. Conclusions

The application of different WA treatments resulted in varying degrees of improvement in both yield and quality of *Morchella*, with the WA2 group showing the most significant improvements. Compared to CK, the WA2 group showed an increase in yield, crude protein, crude fat, crude polysaccharides, TN in the fruiting body, TP and TK by 18.36, 5.45, 6.01, 53.19, 7.03, 13.86 and 14.61%, respectively. As a natural fertiliser, WA provides essential nutrients such as K, N, and P to the soil, thereby improving soil fertility. With increasing amounts of WA added, soil pH, AP, AK, TP, and TK showed a continuous upward trend, while SOM, TN, and AN showed an initial increase followed by a decrease. All treatment groups showed a significant improvement in soil fertility compared to CK (*p* < 0.05). The application of WA altered the structure of the soil microbial community, increasing bacterial diversity and decreasing overall fungal diversity. The relative abundance of beneficial microbial communities for *Morchella* growth, such as Acidobacteriota, increased with the addition of WA, while the relative abundance of detrimental microbial communities, such as *Xanthomonadaceae*, decreased. RDA results indicated that pH and TK were the major environmental factors influencing soil bacterial communities, while pH, TK, and TN were the major factors influencing fungal community structure. Pathway analysis using PLA-PM showed that WA application can improve soil fertility by altering soil physicochemical properties, which directly improves the yield and quality of *Morchella*. In addition, changes in soil physicochemical properties can also alter the structure of soil microbial communities, increasing the abundance of beneficial microbial communities and decreasing that of harmful ones, thereby indirectly affecting the yield and quality of *Morchella*. Therefore, soil physicochemical properties are key factors influencing the yield and quality of *Morchella*. In conclusion, this study not only found that the addition of WA can improve the yield and quality of *Morchella*, but also elucidated the underlying mechanisms by which WA improves yield and quality through the regulation of soil physicochemical properties and microbial communities. These findings provide new insights into how to improve the yield of *Morchella* and offer new perspectives for its cultivation on fertile soils.

## Data Availability

The microbiome sequencing data have been hosted at NCBI BioProject under access numbers PRJNA1177259 and PRJNA1177394.

## Supplementary Materials

The following supporting information can be downloaded at: https://www.mdpi.com/article/10.3390/microorganisms12122406/s1, Figure S1: Experimental process diagram; Figure S2: Plowing the ground (A), Forming ridges (B), Covering with black film (C), Covering with plastic film (D), Primitive stage (E), Fruiting stage (F); Figure S3: Bacterial dilution curve (A) and fungal dilution curve (B). CK, WA1, WA2, WA3, and WA4 represent different amounts of plant ash application (0, 2000, 4000, 6000, and 8000 kg/hm^2^); Figure S4: Bacterial Veen diagram (A) and the fungal Veen diagram (B). CK, WA1, WA2, WA3, and WA4 represent different amounts of plant ash application (0, 2000, 4000, 6000, and 8000 kg/hm^2^); Figure S5: The relative abundance heatmap at the bacterial phylum level (A) and family level (B), and the relative abundance heatmap at the fungal phylum level (C) and family level (D); Figure S6: The linear discriminant analysis effect size (LEfSe) branch plot for bacteria (A) and fungi (B); Table S1: Yield and nutrient composition of *Morchella* fruiting bodies in different WA addition groups; Table S2: Soil physical and chemical properties in different WA addition groups; Table S3: Classification standard values for various soil attributes (refer to the Second National Soil Census); Table S4: Integrated soil fertility (IFI) in different WA addition groups.
