# Supplementary material for: Enhancing Morchella Mushroom Yield and Quality Through the Amendment of Soil Physicochemical Properties and Microbial Community with Wood Ash
Source: Microorganisms. 2024 Nov 23;12(12):2406. doi: 10.3390/microorganisms12122406 (PMC11676116; doi:10.3390/microorganisms12122406)
Supplement: Supplementary file 1 [file microorganisms-12-02406-s001.zip › microorganisms-3307302-supplementary.pdf]

Enhancing *Morchella* Mushroom Yield and Quality through the Amendment of Soil Physicochemical Properties and microbial community with Wood Ash

Kai Huang[1,2], Ling Li[1,2], Weijun Wu[1], Kunlun Pu[1,2], Wei Qi[1], Jianzhao Qi[1,2,3*] and Minglei Li[1,2,*]

[1] Center of Edible Fungi, Northwest A&F University, Yangling, 712100, China;
[2] School of Soil and Water Conservation Science and Engineering, Northwest A&F University, Yangling 712100, China;
[3] Shaanxi Key Laboratory of Natural Products & Chemical Biology, College of Chemistry & Pharmacy, Northwest A&F University, Yangling, 712100, China
* Correspondence: Minglei Li, mlli@nwsuaf.edu.cn;
Jianzhao Qi, qjz@nwafu.edu.cn

Table S1: Yield and nutrient composition of *Morchella* fruiting bodies in different WA addition groups. 1
Table S2: Soil physical and chemical properties in different WA addition groups. 2
Table S3: Classification standard values for various soil attributes (refer to the Second National Soil Census). 3
Table S4: Integrated Soil Fertility (IFI) in different WA addition groups. 4
Figure S1. Experimental process diagram. 5
Figure S2. Plowing the ground (A), Forming ridges (B), Covering with black film (C), Covering with plastic film (D), Primitive stage (E), Fruiting stage (F). 6
Figure S3. Bacterial dilution curve (A) and fungal dilution curve (B). 7
Figure S4. Bacterial Veen diagram (A) and the fungal Veen diagram (B). 8
Figure S5. The relative abundance heatmap at the bacterial phylum level (A) and family level (B), and the relative abundance heatmap at the fungal phylum level (C) and family level (D). 9
Figure S6. The linear discriminant analysis effect size branch plot for bacteria (A) and fungi (B). 10

Table S1: Yield and nutrient composition of *Morchella* fruiting bodies in different WA addition groups.

Group	TA (Kg/hm^2)	CP (mg/g)	EE (mg/g)	CPS (mg/g)	FTN (g/Kg)	FTP (g/Kg)	FTK (g/Kg)
**CK**	3066.30e	360.56c	33.4c	34.68d	56.36c	18.44c	27.04d
**WA1**	3693.13d	375.96abc	33.7c	40.51c	62.14a	19.77b	28.21c
**WA2**	6695.53a	380.20a	35.4a	53.13a	60.32b	20.99a	31.00b
**WA3**	6094.87b	370.60ab	34.7b	49.40b	60.53ab	21.19a	31.57b
**WA4**	5491.17c	368.60bc	32.2d	48.23b	58.83c	21.30a	33.09a

Note: CK, WA1, WA2, WA3, and WA4 represent different amounts of plant ash application (0, 2000, 4000, 6000, and 8000 Kg/hm2), respectively. The values in the table represent the average values, and different lowercase letters indicate significant differences between different treatments ($p < 0.05$, multiple comparisons with Tukey HSD test). TA: Production volume; CP: crude protein; EE: Crude fat; CPS: Crude polysaccharides; FTN: Total nitrogen of the fruiting body; FTP: Total phosphorus in the fruiting body; FTK: Total potassium in the fruiting body.

Table S2: Soil physical and chemical properties in different WA addition groups.

Group	pH	SOM (g/Kg)	TN (g/Kg)	AN (g/Kg)	TP (g/Kg)	AP (mg/Kg)	TK (mg/Kg)	AK (mg/Kg)	BD (g/cm^3)	SMC (%)
**CK**	8.17e	18.39d	0.98d	0.64d	20.58b	85.59d	16.06c	152.67d	1.88d	0.23a
**WA1**	8.35d	23.89b	1.09c	0.64c	21.85b	91.76d	19.28b	182.67c	2.07b	0.23a
**WA2**	8.45c	24.57b	1.19b	0.65b	21.88b	98.05c	29.68b	203.33b	2.14a	0.25ab
**WA3**	8.54b	25.96a	1.33a	0.72a	21.93a	102.65b	74.19ab	229.67a	2.15a	0.28b
**WA4**	8.68a	21.69c	1.11c	0.72cd	22.24a	88.00a	79.87a	231.67a	2.02c	0.27b

Note: CK, WA1, WA2, WA3, and WA4 represent different amounts of plant ash application (0, 2000, 4000, 6000, and 8000 Kg/hm2), respectively. The values in the table represent the average values, and different lowercase letters indicate significant differences between different treatments ($p < 0.05$, multiple comparisons with Tukey HSD test). pH: Soil acidity and alkalinity; SOM: soil organic matter; TN: Total nitrogen; AN: alkali-hydrolyzable nitrogen; TP: Total phosphorus; AP: soil available phosphorus; TK: Total potassium; AK: soil available potassium; BD: Soil bulk density; SMC: Soil moisture content.

Table S3: Classification standard values for various soil attributes (refer to the Second National Soil Census).

classification	pH	SOM	TN	AN	TP	AP	TK	AK
**Xa**	4.5	10	0.75	60	0.4	3	5	40
**Xc**	6.5	20	1.5	120	0.6	10	20	100
**Xp**	8.5	30	2	180	1.0	20	2.5	150

(a) Calculation of fertility coefficient IFIi

$$IFIi = \begin{cases} \frac{X}{Xa} & X \le Xa; \\ 1 + \frac{X-Xa}{Xc-Xa} & Xa < X \le Xc\,; \\ 2 + \frac{X-Xa}{Xp-Xc} & Xc < X \le Xp; \\ 3 & X > Xp; \end{cases}$$

(b) Integrated Soil Fertility (IFI):

$$IFI = \sqrt{\frac{{IFIi_{average}}^2 + {IFIi_{minimum}}^2}{2}} \times \left(\frac{n-1}{n}\right)$$

In the formula: IFI is the integrated soil fertility index; $IFIi_{average}$ is the mean fertility coefficient of each component; $IFIi_{minimum}$ is the minimum value of the fertility coefficient for each component; n is the number of evaluation indicators.

Table S4: Integrated Soil Fertility (IFI) in different WA addition groups.

Group	CK	WA1	WA2	WA3	WA4
**IFI**	1.88d	2.07b	2.14a	2.15a	2.02c

Note: The IFI value ranges from 0 to 3, and the larger the IFI value, the higher the overall soil fertility.

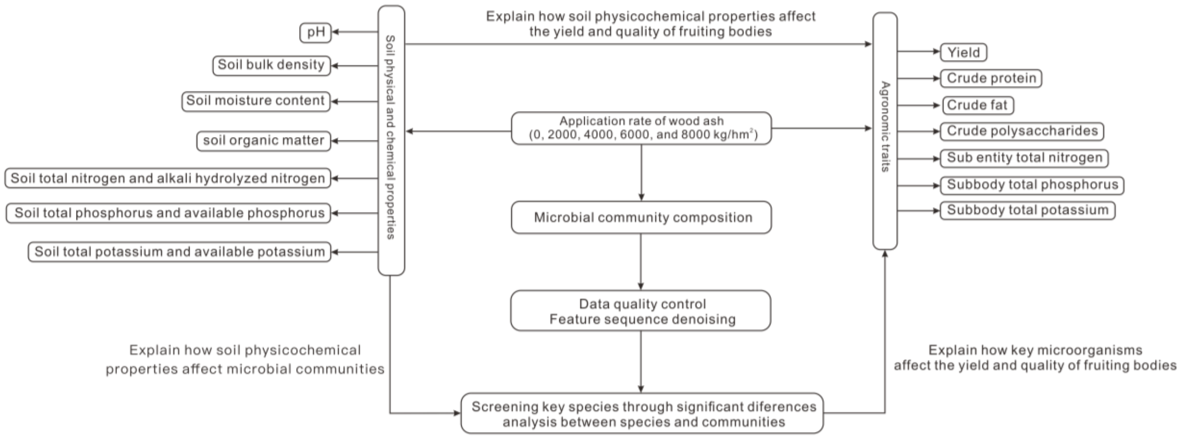

Figure S1. Experimental process diagram.

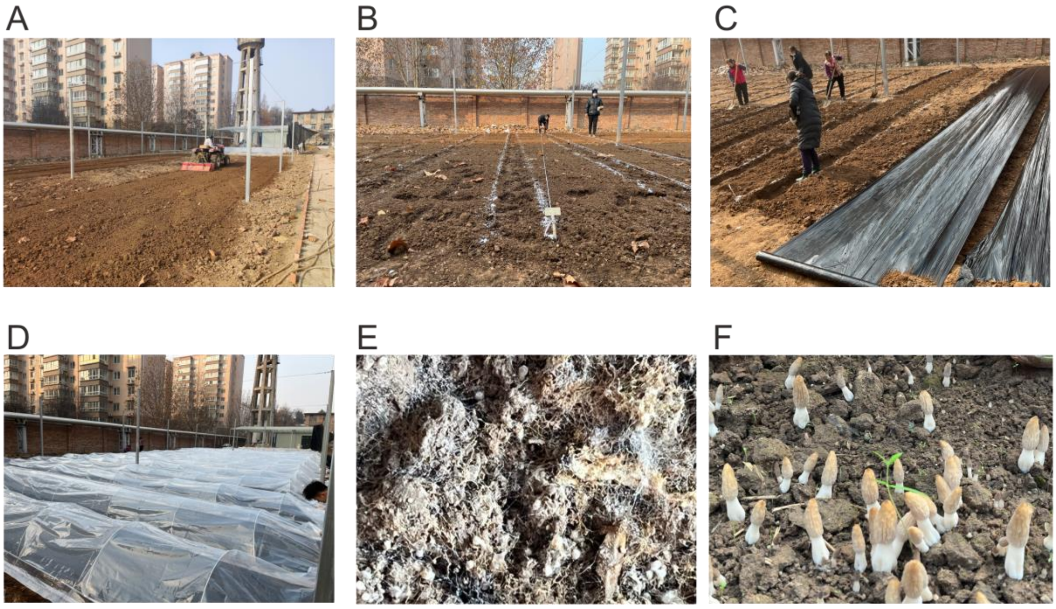

Figure S2. Plowing the ground (A), Forming ridges (B), Covering with black film (C), Covering with plastic film (D), Primitive stage (E), Fruiting stage (F).

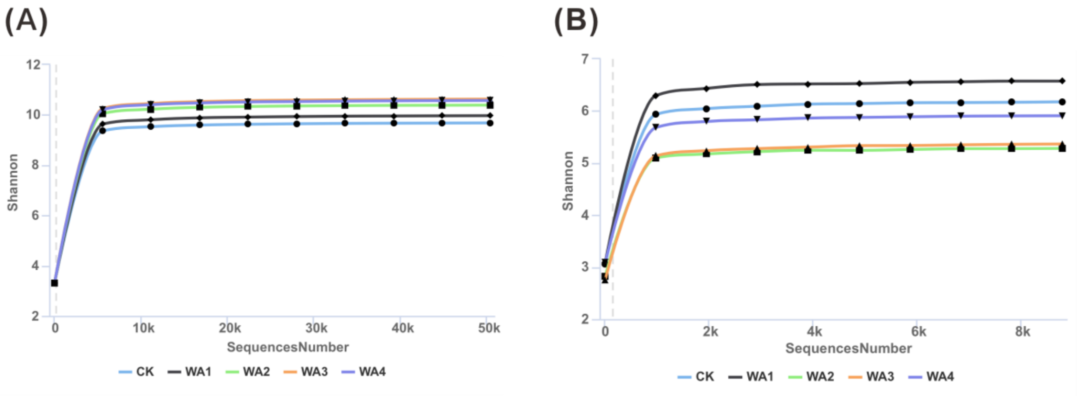

Figure S3. Bacterial dilution curve (A) and fungal dilution curve (B).
CK, WA1, WA2, WA3, and WA4 represent different amounts of plant ash application (0, 2,000, 4,000, 6,000, and 8,000 Kg/hm^2).

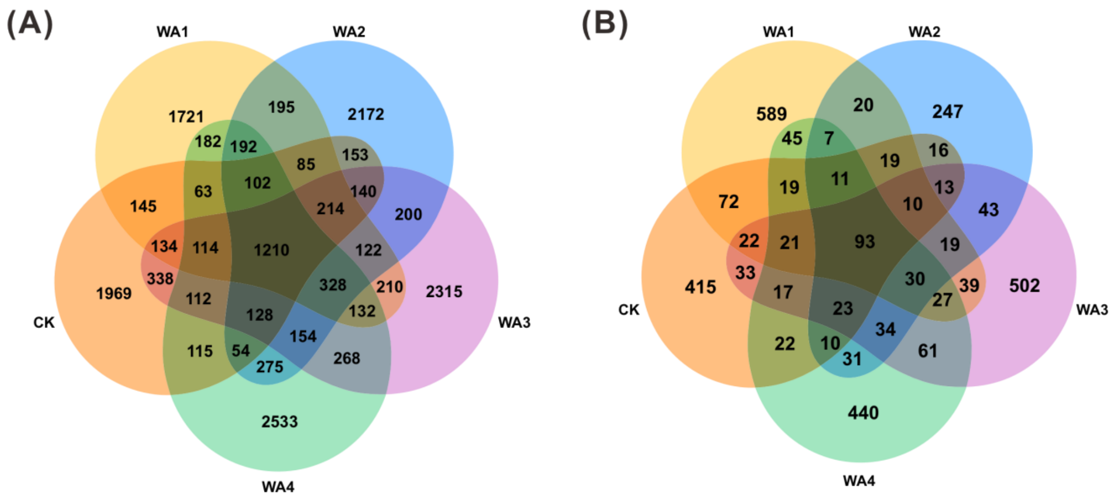

Figure S4. Bacterial Veen diagram (A) and the fungal Veen diagram (B).

CK, WA1, WA2, WA3, and WA4 represent different amounts of plant ash application (0, 2,000, 4,000, 6,000, and 8,000 Kg/hm^2).

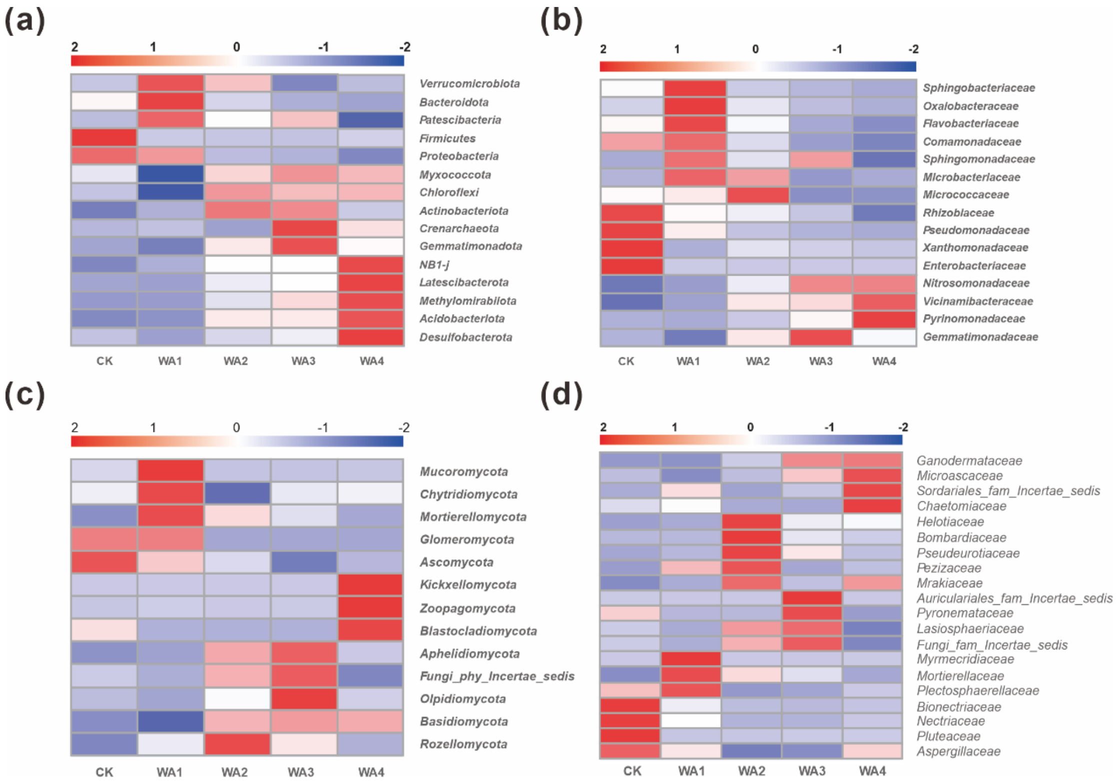

Figure S5. The relative abundance heatmap at the bacterial phylum level (A) and family level (B), and the relative abundance heatmap at the fungal phylum level (C) and family level (D).

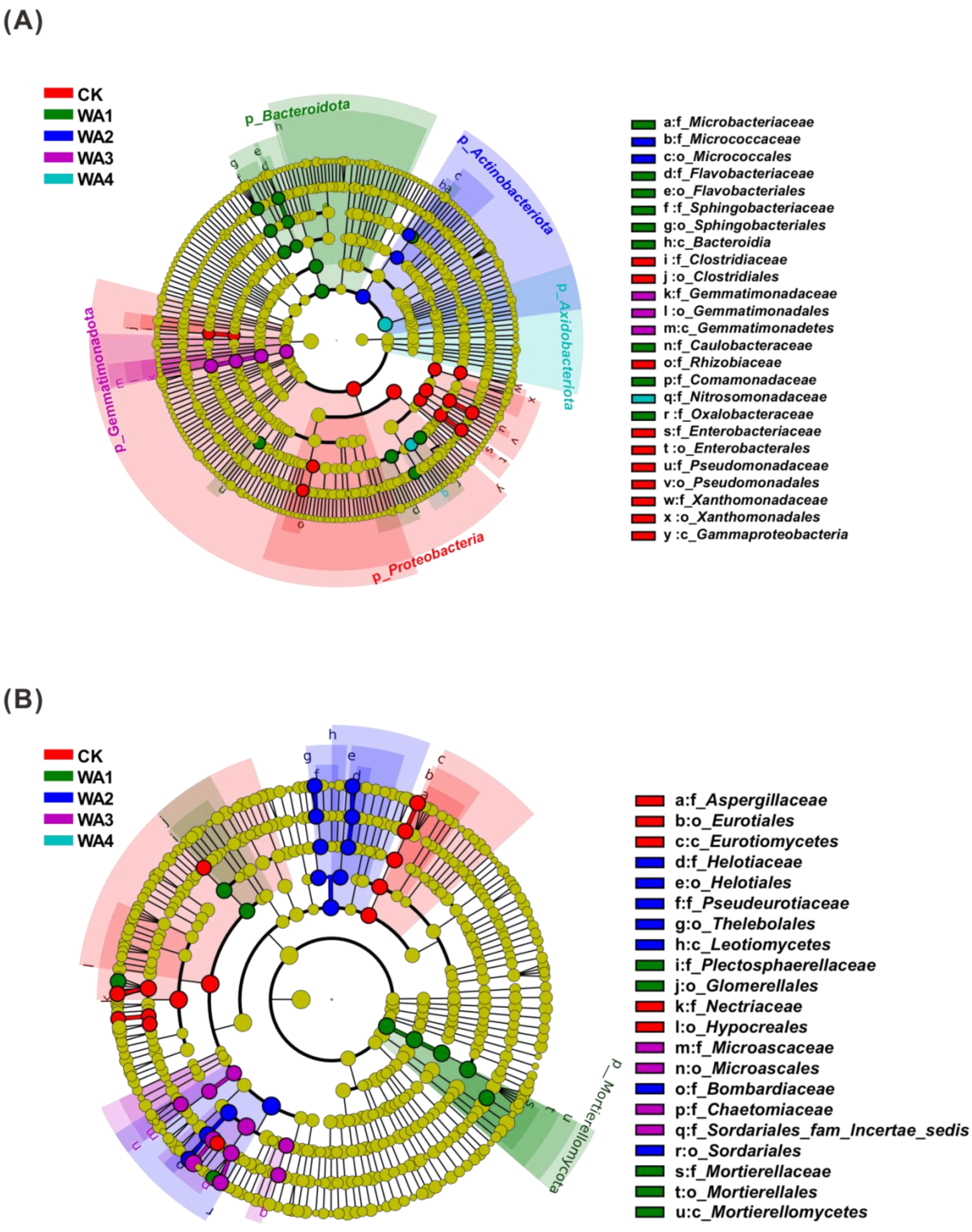

Figure S6. The linear discriminant analysis effect size branch plot for bacteria (A) and fungi (B).
